# Longitudinal pulmonary outcomes after posterior spinal fusion in Duchenne muscular dystrophy: a retrospective cohort study

**DOI:** 10.1007/s00590-026-05024-1

**Published:** 2026-11-12

**Authors:** Rami Rajjoub, James Caid Kirven, Mutaleeb Shobode, Ryan Goodwin, Reid Chambers, Allan Beebe, Daniel DeGenova, Kirsten Tulchin-Francis, Sean Tabaie, Allen Kadado

**Affiliations:** 1 Department of Orthopedics, Nationwide Childre’s Hospital, Columbus, USA; 2 Sonoran Spine Research and Education Foundation, Tempe, USA

**Keywords:** Duchenne muscular dystrophy, Neuromuscular scoliosis, Posterior spinal fusion, Pulmonary function, Forced vital capacity, Scoliosis correction

## Abstract

**Introduction:**

Whether posterior spinal fusion (PSF) alters pulmonary decline in patients with Duchenne muscular dystrophy (DMD) remains uncertain. This study evaluated longitudinal changes in pulmonary function, respiratory muscle strength after PSF, examined whether the magnitude of scoliosis correction was associated with postoperative pulmonary outcomes

**Methods:**

We retrospectively reviewed patients with DMD who underwent PSF and had pulmonary function testing before and after surgery. The primary outcome was forced vital capacity (FVC) percent predicted, assessed preoperatively, at approximately 5–6 months postoperatively, and at final follow-up of approximately 2 years. Measured FVC, the FEV_1_/FVC ratio, and maximal inspiratory (MIP) and expiratory (MEP) pressures were secondary outcomes.

**Results:**

Sixteen nonambulatory male patients were included at a mean age of 17.9 ± 2.9 years. The mean Cobb angle improved from 63.6° ± 15.4° preoperatively to 19.1° ± 13.3° postoperatively. Mean FVC percent predicted was unchanged from the preoperative to the early postoperative assessment (41.0% ± 15.5% vs 41.1% ± 18.0%; *P* = 0.511) but decreased to 34.4% ± 13.7% at final follow-up (mean change, − 8.9 percentage points; *P* = 0.006). MEP decreased during the early postoperative period (*P* = 0.010) and no longer differed from baseline at final follow-up, whereas MIP was unchanged throughout. The magnitude of curve correction was not significantly associated with change in FVC percent predicted at either interval (ρ =  − 0.45, *P* = 0.106 and ρ =  − 0.43, *P* = 0.122). An association in the same direction with measured FVC (ρ =  − 0.62, *P* = 0.019 and ρ =  − 0.65, *P* = 0.012) was confined to the intervals spanning surgery and was absent between the early postoperative and final assessments (ρ =  − 0.18, *P* = 0.565).

**Conclusions:**

In nonambulatory patients with DMD, FVC percent predicted was unchanged early after PSF and declined by approximately 2 years, a trajectory consistent with the natural history of the disease. Greater curve correction was not associated with better pulmonary outcomes. These findings do not support pulmonary preservation as an expected benefit of deformity correction and reinforce the functional, postural, and quality-of-life goals of PSF.

## Introduction

Duchenne muscular dystrophy (DMD) is an X-linked neuromuscular disorder characterized by progressive skeletal, respiratory, and cardiac muscle weakness [1]. Scoliosis develops in many affected individuals, with clinically significant spinal curvature occurring in up to 90% of patients not treated with corticosteroids [2, 3]. Progressive scoliosis may further compromise respiratory mechanics through thoracic distortion and reduced chest-wall compliance [1, 4]. Natural history data from the Cooperative International Neuromuscular Research Group (CINRG) and the Canadian Neuromuscular Disease Registry (CNDR) show that forced vital capacity (FVC) percent predicted plateaus around 10 years of age and then declines steadily, at reported rates of approximately 2.5–6 percentage points per year depending on age, ambulatory status, and glucocorticoid exposure [5–7]. Respiratory insufficiency remains a leading cause of morbidity and mortality in DMD. [4, 5]

Posterior spinal fusions (PSF) are recommended for progressive curves exceeding approximately 20° to 30° in patients with DMD, with the goals of preventing further deformity, restoring sitting balance, and improving comfort and care [8, 9]. Whether PSF also preserves pulmonary function remains uncertain. Several studies have reported slower postoperative declines in FVC percent predicted, and comparative observational studies have suggested improved function or survival among surgically treated patients [4, 10–12]. However, other studies have found that spinal fusion did not alter pulmonary decline, and a recent Cochrane review concluded that the current evidence is insufficient to support respiratory function alone as an indication for surgery. [1, 10]

The association between the magnitude of scoliosis correction and postoperative pulmonary outcomes remains poorly understood. This study aimed to characterize longitudinal changes in pulmonary function and respiratory muscle strength after PSF in patients with DMD and to determine whether the degree of curve correction was associated with postoperative pulmonary decline.

## Methods

### Study design and participants

We performed a retrospective cohort study of patients with DMD and scoliosis who underwent PSF and follow-ups at our institution from September 2011 to June 2025. Patients were eligible for inclusion if they had a confirmed diagnosis of DMD and scoliosis, were treated with PSF, and had documented pulmonary function testing (PFT) both before and after surgery. Patients were excluded if their spinal deformity was attributable to a diagnosis other than DMD (for example, another dystrophinopathy or neuromuscular disorder, or a congenital or syndromic primary skeletal deformity) or if pre- or postoperative PFT data were incomplete.

### Data collection

Demographic and clinical variables were extracted from the electronic medical record, including age at surgery, sex, ambulatory status, and medication exposure, including corticosteroids, bisphosphonates, and exon-skipping or gene therapy. Radiographic variables included the major-curve coronal Cobb angle at initial presentation, the immediately preoperative Cobb angle, and the postoperative residual Cobb angle, defined as the major-curve Cobb angle remaining after instrumented correction, measured on the first upright (seated) radiograph obtained after surgery. Absolute curve correction was calculated as the preoperative Cobb angle minus the postoperative residual Cobb angle, and relative curve correction as the absolute correction divided by the preoperative Cobb angle, expressed as a percentage. Operative variables included the uppermost and lowermost instrumented vertebrae, operative duration, estimated blood loss, length of hospital stay, and intraoperative and postoperative complications.

Pulmonary function was assessed at three time points: preoperatively, at an early postoperative visit approximately 5 to 6 months after surgery, and at final follow-up approximately 2 years after surgery. At each assessment, we recorded measured FVC (L), FVC percent predicted, the FEV_1_/FVC ratio expressed as a percentage of the predicted value, maximal inspiratory pressure (MIP; cm H_2_O), and maximal expiratory pressure (MEP; cm H_2_O). Spirometry and respiratory pressure measurements were performed in the seated position in the institutional pediatric pulmonary function laboratory. Spirometry was performed and graded according to the updated American Thoracic Society/European Respiratory Society (ATS/ERS) standardization of spirometry,^13^ and only maneuvers meeting acceptability and repeatability criteria were reported in the medical record and used in this analysis; no technically unacceptable tests were included. Predicted FVC was derived from the Global Lung Function Initiative 2012 reference equations,^14^ and FVC percent predicted was recalculated for analysis as measured FVC divided by predicted FVC at the same visit. Height was measured by the pulmonary function laboratory with a tape measure at the time of each spirometry test, so that the predicted value at each visit reflected the height recorded at that visit. [15]

### Outcome measures

The primary outcome was the within-patient change in FVC percent predicted across the preoperative-to-early postoperative, preoperative-to-final follow-up, and early postoperative-to-final follow-up intervals. FVC percent predicted was designated the primary measure because it accounts for the age- and height-related change in expected lung volume that occurs across a 2-year interval in adolescents. Measured FVC (L) was analyzed as a secondary outcome. It was retained because it is independent of the height estimate, which is a recognized source of error in nonambulatory patients with contractures and spinal deformity^15^, and because absolute FVC thresholds carry prognostic meaning in DMD [5]. Other secondary outcomes were changes in the FEV_1_/FVC ratio, MIP, and MEP. Exploratory analyses assessed associations between absolute and relative curve correction and changes in FVC percent predicted and measured FVC, and compared changes in pulmonary function according to corticosteroid and bisphosphonate exposure.

### Statistical analysis

Continuous variables are reported as mean ± standard deviation or median with interquartile range, as appropriate, and categorical variables as counts and percentages. Denominators are provided for variables with missing data. Within-patient changes over time were assessed using the Wilcoxon signed-rank test. Exact p values were also calculated and produced no changes in statistical significance. Associations between continuous variables were evaluated using Spearman rank correlation coefficients (ρ). Between-group differences in pulmonary function changes according to corticosteroid and bisphosphonate exposure were assessed using two-sided Mann–Whitney U tests. Because the correlation and subgroup analyses were exploratory and involved multiple comparisons, Holm-adjusted *P* values were additionally calculated across the five pulmonary measures for the primary interval and across the between-group comparisons by medication exposure; unadjusted values are presented in the tables and adjusted values are reported in the text where they alter interpretation. All tests were two-sided, with *p* < 0.05 considered statistically significant. Analyses were performed using Python version 3.11 with NumPy and SciPy.

## Results

### Study population

Nineteen patients with DMD met the initial inclusion criteria, but three were excluded due to lack of operative data. Sixteen patients were included in the analysis (Table 1). All patients were male and nonambulatory at baseline, with a mean age at surgery of 17.9 ± 2.9 years. The mean Cobb angle at presentation was 29.0° ± 21.5°, and curves progressed by a mean of 34.5° ± 25.3° during the 2.95 ± 2.37 years between presentation and surgery. Corticosteroid therapy was documented in 14 patients (87.5%). Of the 15 patients with available medication data, 8 (53.3%) had received bisphosphonate therapy. No patient had received exon-skipping or gene-transfer therapy.

**Table 1:** Baseline demographic, radiographic, and pharmacologic characteristics of the study cohort (n = 16, unless specified)

Characteristic	Mean ± SD or n (%)
Age at surgery, years	17.9 ± 2.9
Male sex	16/16 (100.0)
Non-ambulatory at baseline	16/16 (100.0)
Cobb angle at presentation, °	29.0 ± 21.5
Curve progression, presentation to surgery, °	34.5 ± 25.3
Presentation to surgery, years	2.95 ± 2.37
*Corticosteroid therapy*	
Yes	14 (87.5)
No	2 (12.5)
*Bisphosphonate therapy (n* = *15)*	
Yes	8 (53.3)
No	7 (46.7)
*Disease-modifying therapy (exon skipping or gene transfer)*	
No	16 (100.0)

Values are mean ± standard deviation for continuous variables and n (%) for categorical variables. All patients carried a clinically confirmed diagnosis of Duchenne muscular dystrophy, and all were non-ambulatory at baseline and remained so postoperatively. Cobb angle at presentation refers to the major curve measured on the first available upright or seated radiograph; curve progression is the difference between the Cobb angle at presentation and the immediate preoperative Cobb angle

### Operative characteristics

Operative characteristics are summarized in Table 2. The mean Cobb angle improved from 63.6° ± 15.4° preoperatively to 19.1° ± 13.3° postoperatively, corresponding to an absolute correction of 44.4° ± 17.1° and a relative correction of 69.6% ± 22.6%. Mean operative time was 6.5 ± 1.4 h, estimated blood loss was 1022 ± 410 mL, and hospital length of stay was 7.0 ± 3.5 days.

**Table 2:** Operative, radiographic, and perioperative outcomes following posterior spinal fusion (n = 16, unless specified)

Variable	Mean ± SD or n (%)
Cobb angle, pre-operative, °	63.6 ± 15.4
Cobb angle, post-operative residual, °	19.1 ± 13.3
Absolute curve correction, °	44.4 ± 17.1
Relative curve correction, %	69.6 ± 22.6
Operative time, hours	6.5 ± 1.4
Estimated blood loss, mL	1022 ± 410
Length of hospital stay, days	7.0 ± 3.5
*Upper instrumented vertebra*	
T2	12 (75.0)
T3	4 (25.0)
*Lowest instrumented vertebra*	
S1	7 (43.8)
S2 (including S2-alar-iliac)	3 (18.8)
Pelvis / ilium	6 (37.5)
*Pelvic fixation*	
Yes	13 (81.2)
No	3 (18.8)
*Intra-operative complication (n* = *13)*	
No	13 (100.0)
*Post-operative complication (n* = *13)*	
No	11 (84.6)
Yes	2 (15.4)

Values are mean ± standard deviation or n (%). Pre-operative and post-operative residual Cobb angles refer to the major curve; the residual angle was measured on the first upright (seated) radiograph after surgery. Absolute curve correction is the difference between the preoperative and post-operative residual Cobb angle, and relative correction is that difference expressed as a percentage of the preoperative Cobb angle

The upper instrumented vertebra was T2 in 12 patients (75.0%) and T3 in 4 (25.0%). The lowest instrumented level was S1 in 7 patients (43.8%), S2 or S2-alar-iliac in 3 (18.8%), and the pelvis or ilium in 6 (37.5%). Overall, pelvic fixation was used in 13 patients (81.2%). Among the 13 patients with available complication data, no intraoperative complications occurred and 2 (15.4%) experienced postoperative complications, including one patient who developed urinary retention, and another patient who sustained a periprosthetic femur fracture followed by pulmonary embolism and duodenal ulcer.

### Pulmonary function across the perioperative course

**Fig. 1:**
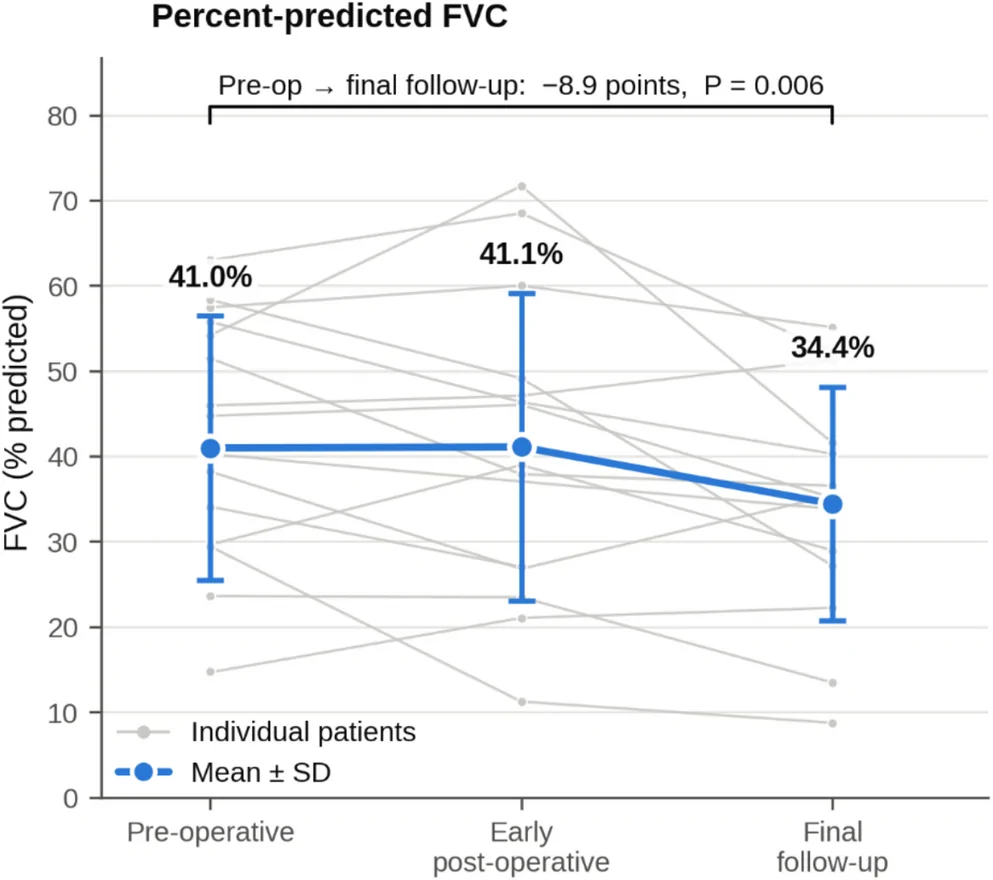
Percent-predicted forced vital capacity across the perioperative course. Percent-predicted FVC in individual patients (grey lines) and cohort mean ± SD (blue) at the pre-operative (n = 16), early post-operative (n = 14), and final follow-up (n = 14) assessments. Mean percent-predicted FVC was unchanged between the pre-operative and early post-operative assessments (41.0% vs 41.1%) and declined by final follow-up (34.4%; mean change from pre-operative to final follow-up, − 8.9 percentage points; *P* = 0.006). FVC = forced vital capacity; SD = standard deviation

**Fig. 2:**
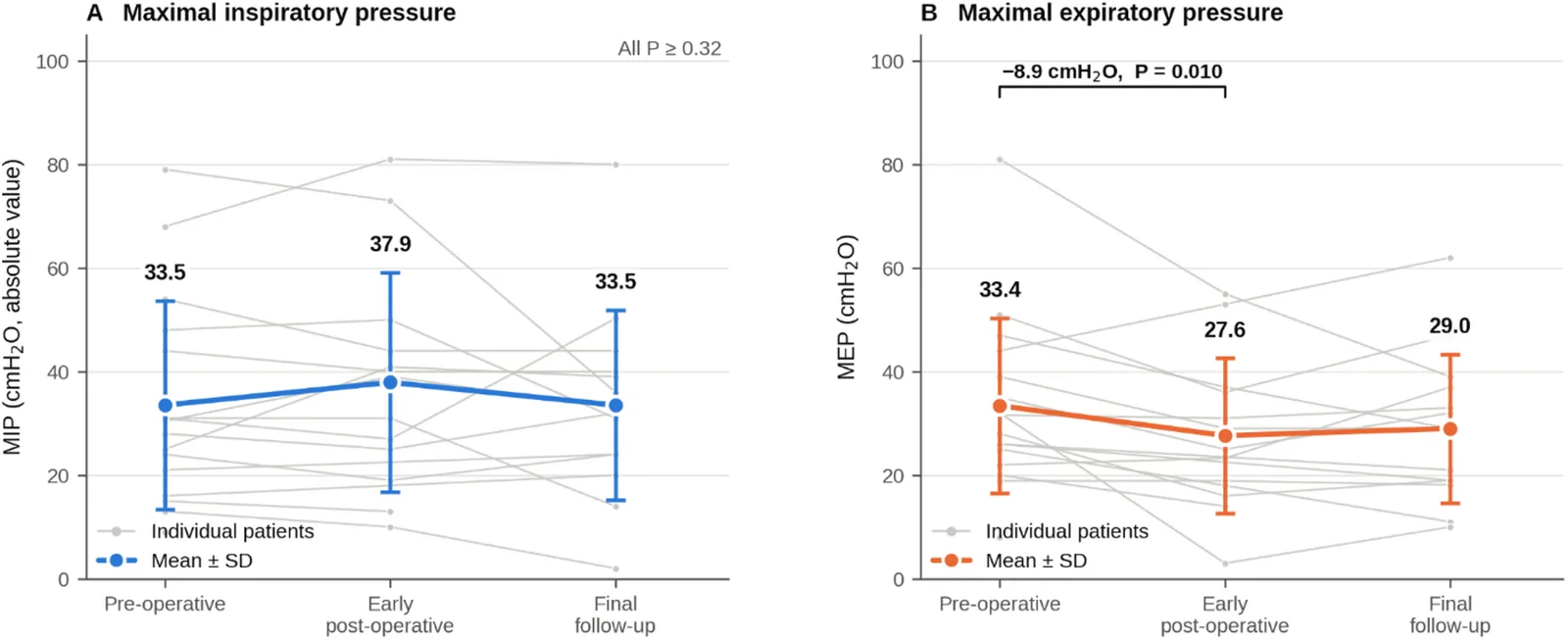
Respiratory muscle strength across the perioperative course. **A** Maximal inspiratory pressure (MIP, absolute value) at the pre-operative, early post-operative, and final follow-up assessments. MIP did not change significantly at any interval. **B** Maximal expiratory pressure (MEP) at the same assessments. MEP fell between the pre-operative and early post-operative assessments (*P* = 0.010) and was 29.0 cm H_2_O at final follow-up, not significantly different from either earlier value

Pulmonary function and respiratory muscle strength at each assessment are presented in Table 3 and Figs. 1 and 2. Mean FVC percent predicted was 41.0% ± 15.5% preoperatively, 41.1% ± 18.0% at early postoperative follow-up, and 34.4% ± 13.7% at final follow-up (Fig. 1). Corresponding measured FVC values, reported descriptively, were 1.47 ± 0.60 L, 1.44 ± 0.66 L, and 1.35 ± 0.71 L.

**Table 3:** Pulmonary function at the three prespecified study time points

Variable	Pre-operative	Early post-operative	Final follow-up
FVC, % predicted (primary outcome)	41.0 ± 15.5	41.1 ± 18.0	34.4 ± 13.7
FVC, L	1.47 ± 0.60	1.44 ± 0.66	1.35 ± 0.71
FEV_1_/FVC ratio, % of predicted	101.4 ± 13.1	99.1 ± 19.2	99.5 ± 13.4
Maximal inspiratory pressure, cm H_2_O	33.5 ± 20.1	37.9 ± 21.2	33.5 ± 18.3
Maximal expiratory pressure, cm H_2_O	33.4 ± 16.9	27.6 ± 15.0	29.0 ± 14.3

Values are mean ± standard deviation. FVC percent predicted was the primary outcome; measured FVC in liters is reported as a secondary, descriptive measure. FEV_1_/FVC is expressed as percent of predicted; maximal inspiratory and maximal expiratory pressures are reported in cm H_2_O. Pre-operative n = 16; early post-operative and final follow-up n = 14 for spirometry (n = 13 and 14, respectively, for respiratory pressures)

The mean FEV_1_/FVC ratio, expressed as a percentage of the predicted value, was 101.4% ± 13.1% preoperatively, 99.1% ± 19.2% at early follow-up, and 99.5% ± 13.4% at final follow-up. Mean MIP was 33.5 ± 20.1 cm H_2_O preoperatively, 37.9 ± 21.2 cm H_2_O at early follow-up, and 33.5 ± 18.3 cm H_2_O at final follow-up (Fig. 2A). Mean MEP was 33.4 ± 16.9 cm H_2_O, 27.6 ± 15.0 cm H_2_O, and 29.0 ± 14.3 cm H_2_O at the respective assessments (Fig. 2B).

### Within-patient changes in pulmonary function

Within-patient changes across each study interval are presented in Table 4. From the preoperative to early postoperative assessment, FVC percent predicted did not change (− 1.8 ± 10.0 percentage points; *P* = 0.511). MEP decreased by 8.9 ± 10.5 cm H_2_O (effect size r =  − 0.80, *P* = 0.010), whereas MIP was unchanged (0.2 ± 7.7 cm H_2_O; *P* = 0.931). Changes in the remaining measures were not significant.

**Table 4:** Within-patient change in pulmonary function after posterior spinal fusion

Variable	Mean change ± SD	r	p-value
*Pre-operative to early post-operative*			
FVC, % predicted	− 1.8 ± 10.0	− 0.20	0.511
FVC, L	− 0.10 ± 0.27	− 0.39	0.175
FEV_1_/FVC ratio, % of predicted	− 2.6 ± 17.3	− 0.06	0.587
Maximal inspiratory pressure, cm H_2_O	0.2 ± 7.7	− 0.14	0.931
Maximal expiratory pressure, cm H_2_O	− 8.9 ± 10.5	− 0.80	0.010
*Pre-operative to final follow-up*			
FVC, % predicted	− 8.9 ± 10.2	− 0.81	0.006*
FVC, L	− 0.20 ± 0.38	− 0.59	0.070
FEV_1_/FVC ratio, % of predicted	− 4.5 ± 16.2	− 0.20	0.319
Maximal inspiratory pressure, cm H_2_O	− 3.1 ± 15.9	− 0.12	0.481
Maximal expiratory pressure, cm H_2_O	− 7.2 ± 14.9	− 0.55	0.095
*Early post-operative to final follow-up*			
FVC, % predicted	− 7.7 ± 10.7	− 0.74	0.023
FVC, L	− 0.12 ± 0.37	− 0.34	0.249
FEV_1_/FVC ratio, % of predicted	− 1.1 ± 21.0	− 0.21	0.856
Maximal inspiratory pressure, cm H_2_O	− 4.6 ± 15.0	− 0.38	0.317
Maximal expiratory pressure, cm H_2_O	1.7 ± 8.7	0.27	0.502

Values are mean change ± standard deviation, with negative values indicating decline. FVC percent predicted was the primary outcome; measured FVC (L) was a secondary outcome. Effect size r was calculated as the standardized Wilcoxon test statistic divided by the square root of the number of paired observations. p-values correspond to the paired within-patient comparison across the interval. *Holm-adjusted *P* = 0.026 across the five pulmonary measures for this interval

From the preoperative assessment to final follow-up, FVC percent predicted decreased by 8.9 ± 10.2 percentage points (r =  − 0.81, *P* = 0.006; Holm-adjusted *P* = 0.026). Measured FVC decreased by 0.20 ± 0.38 L, although this difference did not reach significance (r =  − 0.59, *P* = 0.070). No significant changes were observed in the FEV_1_/FVC ratio, MIP, or MEP.

Between the early postoperative assessment and final follow-up, FVC percent predicted decreased by 7.7 ± 10.7 percentage points (r =  − 0.74, *P* = 0.023), indicating that most of the decline over the study period accrued after the early postoperative assessment. Changes in measured FVC, the FEV_1_/FVC ratio, MIP, and MEP were not significant.

### Associations between curve correction and pulmonary function

Associations between clinical and operative variables and changes in pulmonary function from the preoperative assessment to final follow-up are presented in Table 5. The change in FVC percent predicted was not significantly associated with age at surgery, preoperative Cobb angle, absolute curve correction (ρ =  − 0.433, *P* = 0.122), relative curve correction (ρ =  − 0.341, *P* = 0.233), preoperative FVC percent predicted, estimated blood loss, or operative time (all *P* ≥ 0.122).

**Table 5:** Spearman rank correlations between preoperative, radiographic, and operative covariates and the change in pulmonary function from the preoperative assessment to final follow-up (n = 14)

Covariate	Spearman ρ	p-value
*Change in FVC % predicted (primary outcome), pre-operative to final follow-up*		
Age at surgery, years	0.222	0.446
Pre-operative Cobb angle, °	− 0.209	0.474
Absolute curve correction, °	− 0.433	0.122
Relative curve correction, %	− 0.341	0.233
Pre-operative FVC, % predicted	− 0.411	0.144
Estimated blood loss, mL	0.131	0.656
Operative time, hours	− 0.204	0.503
*Change in FVC (L) (secondary outcome), pre-operative to final follow-up*		
Age at surgery, years	0.059	0.840
Pre-operative Cobb angle, °	− 0.503	0.067
Absolute curve correction, °	− 0.648	0.012
Relative curve correction, %	− 0.253	0.383
Pre-operative FVC, % predicted	− 0.218	0.455
Estimated blood loss, mL	0.104	0.724
Operative time, hours	− 0.192	0.529

Values are the Spearman correlation coefficient (ρ) with the corresponding unadjusted p-value; negative coefficients indicate that higher values of the covariate were associated with greater pulmonary decline. These analyses were exploratory

For the secondary outcome of measured FVC, greater absolute curve correction was associated with a greater decrease from the preoperative assessment to final follow-up (ρ =  − 0.648, *P* = 0.012). A nonsignificant association in the same direction was present for the preoperative Cobb angle (ρ =  − 0.503, *P* = 0.067), which is closely related to absolute correction by construction. Change in measured FVC was not significantly associated with age at surgery, relative curve correction, preoperative FVC percent predicted, estimated blood loss, or operative time.

**Fig. 3:**
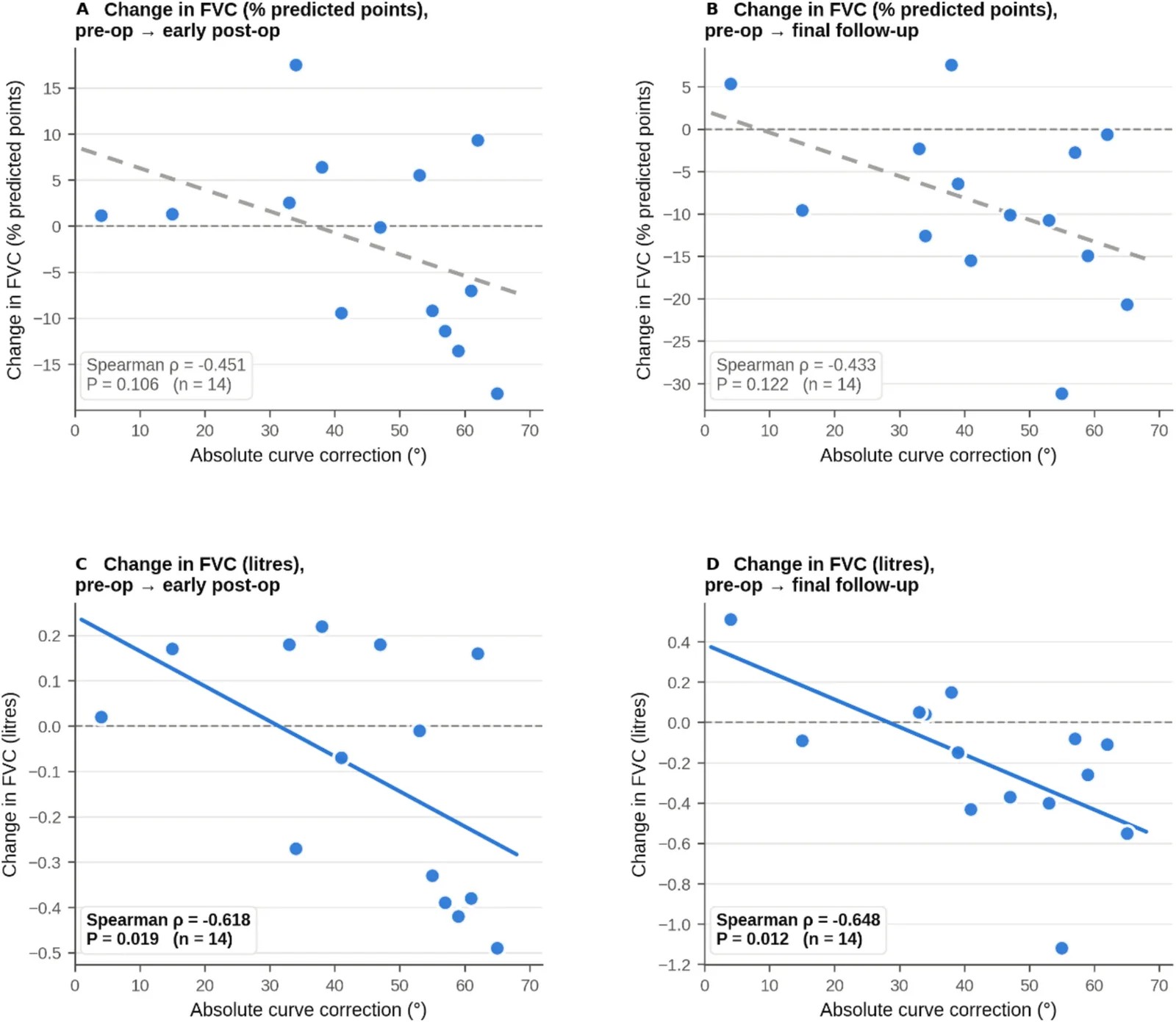
Association between curve correction and change in pulmonary function (n = 14). Absolute curve correction (pre-operative minus post-operative residual Cobb angle) plotted against within-patient change in FVC percent predicted (**A**, **B**; primary outcome) and measured FVC in liters (**C**, **D**; secondary outcome), from the pre-operative assessment to the early post-operative assessment (**A**, **C**) and to final follow-up (**B**, **D**). Lines are least-squares fits, solid where the Spearman correlation reached significance and dashed where it did not. The association with change in FVC percent predicted was negative at both intervals but did not reach significance (ρ =  − 0.45, *P* = 0.106; ρ =  − 0.43, *P* = 0.122). The association with change in measured FVC was in the same direction and reached significance at both intervals (ρ =  − 0.62, *P* = 0.019; ρ =  − 0.65, *P* = 0.012). Neither measure was associated with correction over the interval from early post-operative assessment to final follow-up (|ρ| ≤ 0.18, all *P* ≥ 0.56)

Interval-specific associations are illustrated in Fig. 3. For FVC percent predicted, the association with absolute curve correction was negative at both the preoperative to early postoperative interval (ρ =  − 0.45, *P* = 0.106; Fig. 3A) and the preoperative to final follow-up interval (ρ =  − 0.43, *P* = 0.122; Fig. 3B), but neither reached significance. For measured FVC, the corresponding associations were of the same direction and larger magnitude and reached significance at both intervals (ρ =  − 0.62, *P* = 0.019, Fig. 3C; ρ =  − 0.65, *P* = 0.012, Fig. 3D). Greater relative curve correction was also associated with a greater early postoperative decrease in measured FVC (ρ =  − 0.68, *P* = 0.008), but this association was not maintained at final follow-up. Neither absolute nor relative curve correction was associated with changes in measured FVC or FVC percent predicted between the early postoperative assessment and final follow-up (|ρ| ≤ 0.18; all *P* ≥ 0.56), indicating that any correction-associated difference was established across the operative interval and did not widen during subsequent follow-up.

### Exploratory subgroup analyses by corticosteroid and bisphosphonate exposure

Changes in pulmonary function stratified by corticosteroid and bisphosphonate exposure are presented in Table 6. No significant differences were observed between patients who had and had not received corticosteroid therapy in the change in FVC, FVC percent predicted, FEV_1_/FVC ratio, MIP, or MEP during any study interval (all *P* ≥ 0.132).

**Table 6:** Change in pulmonary function by pre-operative corticosteroid (A) and bisphosphonate (B) exposure (exploratory analysis)

Time interval and variable	Median change [IQR]	Median change [IQR]	p-value
A. Corticosteroid use	Exposed	Unexposed	
*Pre-operative to early post-operative*			
FVC, L	− 0.04 [ − 0.38 to 0.16]	− 0.05 [ − 0.16 to 0.07]	0.659
FVC, % predicted	− 3.6 [ − 9.9 to 2.3]	10.0 [6.3 to 13.8]	0.132
FEV_1_/FVC ratio, % predicted	3.5 [ − 10.2 to 8.2]	− 0.5 [ − 4.8 to 3.8]	1.000
Maximal inspiratory pressure, cm H_2_O	− 3.0 [ − 4.5 to 1.0]	2.3 [ − 0.8 to 5.4]	0.769
Maximal expiratory pressure, cm H_2_O	− 10.0 [ − 13.5 to − 6.5]	− 0.3 [ − 0.4 to − 0.2]	0.231
*Pre-operative to final follow-up*			
FVC, L	− 0.21 [ − 0.41 to − 0.09]	0.04 [0.04 to 0.05]	0.198
FVC, % predicted	− 9.9 [ − 15.1 to − 2.2]	− 7.5 [ − 10.0 to − 4.9]	0.923
FEV_1_/FVC ratio, % predicted	0.5 [ − 10.8 to 5.5]	0.0 [ − 1.5 to 1.5]	0.923
Maximal inspiratory pressure, cm H_2_O	− 2.0 [ − 12.5 to 4.0]	10.9 [6.7 to 15.1]	0.264
Maximal expiratory pressure, cm H_2_O	− 8.0 [ − 15.0 to − 3.8]	0.3 [ − 0.2 to 0.9]	0.198
*Early post-operative to final follow-up*			
FVC, L	− 0.26 [ − 0.38 to 0.05]	0.04 [0.04 to 0.05]	0.513
FVC, % predicted	− 6.0 [ − 10.4 to − 0.1]	− 7.5 [ − 10.0 to − 4.9]	0.410
FEV_1_/FVC ratio, % predicted	0.0 [ − 10.5 to 2.0]	0.0 [ − 1.5 to 1.5]	0.769
Maximal inspiratory pressure, cm H_2_O	− 1.4 [ − 14.8 to 0.0]	10.9 [6.7 to 15.1]	0.485
Maximal expiratory pressure, cm H_2_O	5.0 [ − 5.2 to 8.5]	0.3 [ − 0.2 to 0.9]	0.606

B. Bisphosphonate use	Exposed	Unexposed	
*Pre-operative to early post-operative*			
FVC, L	− 0.39 [ − 0.41 to − 0.15]	0.17 [ − 0.13 to 0.18]	0.014†
FVC, % predicted	− 10.4 [ − 13.0 to − 7.6]	1.3 [0.5 to 4.5]	0.035†
FEV_1_/FVC ratio, % predicted	4.5 [ − 7.2 to 8.8]	6.0 [ − 6.0 to 7.5]	0.975
Maximal inspiratory pressure, cm H_2_O	− 3.5 [ − 4.8 to 2.2]	0.0 [ − 3.5 to 10.8]	0.193
Maximal expiratory pressure, cm H_2_O	− 11.0 [ − 14.2 to 10.0]	− 0.6 [ − 8.5 to 0.6]	0.072
*Pre-operative to final follow-up*			
FVC, L	− 0.33 [ − 0.42 to 0.18]	0.04 [ − 0.23 to 0.10]	0.138
FVC, % predicted	− 12.8 [ − 15.3 to 7.5]	− 9.6 [ − 11.4 to 1.5]	0.295
FEV_1_/FVC ratio, % predicted	0.5 [ − 6.0 to 1.8]	3.0 [ − 9.5 to 9.0]	0.628
Maximal inspiratory pressure, cm H_2_O	− 7.0 [ − 10.8 to 1.0]	4.0 [ − 7.2 to 13.0]	0.279
Maximal expiratory pressure, cm H_2_O	− 9.5 [ − 16.0 to 6.0]	− 0.8 [ − 8.5 to 8.2]	0.138
*Early post-operative to final follow-up*			
FVC, L	− 0.06 [ − 0.36 to 0.16]	− 0.13 [ − 0.41 to 0.12]	0.792
FVC, % predicted	− 2.5 [ − 6.0 to 1.4]	− 10.0 [ − 16.4 to − 1.9]	0.432
FEV_1_/FVC ratio, % predicted	1.0 [ − 8.0 to 1.0]	− 4.0 [ − 9.0 to 3.0]	0.721
Maximal inspiratory pressure, cm H_2_O	0.0 [ − 8.0 to 0.0]	− 1.9 [ − 11.5 to 3.0]	0.965
Maximal expiratory pressure, cm H_2_O	3.0 [0.0 to 7.0]	2.0 [ − 3.9 to 8.0]	0.909

Values are median change [interquartile range] within each exposure group for the three study intervals, with negative values indicating decline. p-values are unadjusted two-sided Mann–Whitney U tests. Corticosteroid groups comprised 14 exposed and 2 unexposed patients; bisphosphonate groups comprised 8 exposed and 7 unexposed patients (exposure undocumented in 1). †Not significant after Holm adjustment across the between-group comparisons (smallest adjusted *P* = 0.210). The bisphosphonate-exposed group was operated on later in the series than the unexposed group, and all bisphosphonate-exposed patients had also received corticosteroids

The comparison by bisphosphonate exposure differed by study interval. Over the preoperative to early postoperative interval only, patients with documented bisphosphonate exposure had a greater decrease in measured FVC than those without (median, − 0.39 L [IQR, − 0.41 to − 0.15] vs 0.17 L [IQR, − 0.13 to 0.18]; unadjusted *P* = 0.014) and in FVC percent predicted (median, − 10.4 percentage points [IQR, − 13.0 to − 7.6] vs 1.3 percentage points [IQR, 0.5 to 4.5]; unadjusted *P* = 0.035). The between-group difference in early MEP change did not reach significance. Over the two longer intervals, the preoperative assessment to final follow-up and the early postoperative assessment to final follow-up, no pulmonary measure differed between groups (all *P* ≥ 0.138): the unexposed group declined over the later interval while the exposed group was stable, so that by final follow-up the two groups had reached similar values. None of the between-group comparisons remained significant after Holm adjustment for multiplicity (smallest adjusted *P* = 0.210). Patients with bisphosphonate exposure were also operated on later in the series than unexposed patients, and all bisphosphonate-exposed patients had received corticosteroids.

## Discussion

The effect of PSF on pulmonary function in DMD remains one of the most debated topics in neuromuscular scoliosis management. In this retrospective cohort of 16 nonambulatory males with DMD who underwent PSF, FVC percent predicted was unchanged in the early postoperative period (41.0–41.1%) and declined by final follow-up at approximately 2 years (34.4%, *P* = 0.006), with most of the decline accruing after the early postoperative assessment. The magnitude of curve correction was not associated with change in FVC percent predicted. A same-direction association between correction and the secondary outcome of measured FVC was confined to the intervals spanning surgery and did not widen thereafter. MEP decreased transiently early after surgery, whereas MIP was unchanged at every interval. Taken together, these findings indicate that correction of spinal deformity neither prevented nor demonstrably accelerated the respiratory deterioration characteristic of DMD, and that greater correction did not confer pulmonary benefit.

### Pulmonary function after posterior spinal fusion

The early stability of FVC percent predicted followed by a subsequent decline is broadly consistent with previous studies suggesting that pulmonary function may stabilize temporarily after PSF before the underlying respiratory decline of DMD resumes. Velasco et al. reported that the annual decline in FVC percent predicted decreased from 4.0% before surgery to 1.75% after surgery [11]. Yang et al. similarly observed an initial postoperative increase in FVC followed by subsequent decline, although deterioration was slower among surgically treated patients than among nonsurgical controls [9]. Chua et al. and Suk et al. also reported slower postoperative pulmonary decline among surgically treated patients. [16, 17]

Conversely, other studies have not demonstrated a meaningful pulmonary benefit from PSF. Alexander et al. found no reduction in the rate of FVC decline after surgery and no association between scoliosis severity and respiratory dysfunction [18]. In the CNDR cohort, the annual decline in vital capacity did not differ between patients with and without scoliosis [7]. A Cochrane review likewise concluded that the available evidence was insufficient to establish whether scoliosis surgery preserves respiratory function in DMD [10]. Differences across studies likely reflect variation in age at surgery, baseline respiratory function, corticosteroid exposure, follow-up duration, and the methods used to quantify pulmonary decline.

Our findings are consistent with the natural history of DMD. In the CINRG natural history study and the CNDR, FVC percent predicted declines steadily through the second decade, and the decline of approximately 9 percentage points over roughly 2 years observed here lies within the range those cohorts report for nonambulatory adolescents [5–7, 12, 19]. Patients in the present cohort underwent surgery at a mean age of 17.9 years with a mean preoperative FVC percent predicted of 41.0%, placing them at a stage when continued respiratory deterioration would be expected irrespective of surgical treatment. Therefore, the decline observed at final follow-up most plausibly reflects disease progression rather than an adverse effect of PSF [12, 20]. Importantly, this study evaluated pulmonary function at discrete perioperative assessments and did not compare longitudinal rates of decline before and after surgery.

### Measured versus percent-predicted FVC

The association between curve correction and pulmonary change reached significance for measured FVC but not for FVC percent predicted. We do not interpret this as a discordance between the two measures. The correlations with FVC percent predicted were in the same negative direction at both intervals (ρ =  − 0.45 and − 0.43) and, in a cohort of 14 complete pairs, fell short of significance rather than reversing. Across the whole cohort the two measures moved together, and FVC percent predicted in fact declined more than measured FVC because predicted FVC rose with age over the study period. The weaker correlation for percent predicted is the expected consequence of dividing a measured volume by a predicted value that is itself estimated with error. Predicted FVC depends on height, which in nonambulatory patients with contractures and spinal deformity must be estimated from a surrogate, and different surrogates yield materially different predicted values in the same patient [15]. Any visit-to-visit inconsistency in the height estimate, including a change in apparent trunk length after correction of the deformity, adds variance to percent predicted that is absent from the measured volume [21]. For this reason we retained measured FVC as a secondary outcome while basing our primary conclusions on FVC percent predicted.

### Curve correction and pulmonary function

Greater absolute curve correction was associated with a greater reduction in measured FVC across the operative interval, and this difference was still detectable at 2 years. Three features of the data argue against interpreting this as a dose-dependent harm of correction. First, the association was absent over the interval from early postoperative assessment to final follow-up, indicating a one-time perioperative offset rather than an accelerated trajectory. Second, absolute correction is determined largely by the preoperative curve magnitude, which showed a nonsignificant association of the same direction with change in measured FVC; with 14 patients the two cannot be separated, and regression to the mean operates in the same direction. Third, the association was not significant for FVC percent predicted, the primary outcome. Absolute correction may therefore be better understood as a marker of deformity severity, surgical complexity, or overall disease burden than as a causal exposure. [4, 22, 23]

Whether a causal relationship between the extent of fusion and a small, fixed loss of volume could exist deserves comment. Posterior instrumentation from T2 to the pelvis fixes the thoracic and lumbar spine and may reduce the small contribution of trunk and rib-cage motion to vital capacity, and residual postoperative pain or altered truncal mechanics could reduce effort on early testing [8, 24]. Correction also lengthens the trunk, which would, if anything, raise the predicted volume rather than lower the measured one. Our data cannot adjudicate among these possibilities, and the magnitude of any such effect was modest relative to the disease-related decline. What the data do establish is that greater correction did not yield greater pulmonary benefit, contrary to the assumption in the idiopathic scoliosis literature that correction of the thoracic deformity improves respiratory function [23, 25, 26]. Kim et al. recently showed that the relationship between deformity and pulmonary impairment is attenuated in neuromuscular scoliosis compared with idiopathic and congenital subtypes [23], consistent with the view that in DMD the deformity is a consequence of the underlying weakness rather than an independent driver of respiratory loss.

### Respiratory muscle strength

MEP decreased during the early postoperative period and had returned toward baseline by final follow-up, whereas MIP did not change at any interval. The stability of MIP is informative. It argues against an acute surgically induced loss of inspiratory muscle strength and therefore against inspiratory weakness as the explanation for the early correction-associated difference in measured FVC. The transient fall in MEP is more plausibly explained by factors specific to forced expiration: expiratory pressure depends substantially on abdominal and truncal muscle function, which is affected by incisional pain, deconditioning, and the altered truncal mechanics of a newly instrumented spine [8, 24]. Neither inspiratory nor expiratory strength improved above preoperative values, and over the full study period both measures were stable in the presence of a significant fall in FVC percent predicted. We note that MIP and MEP are maximal, effort-dependent static pressures that may be insensitive to the gradual loss of respiratory muscle endurance that accompanies disease progression in DMD; the dissociation between stable pressures and falling FVC percent predicted in this cohort should therefore be interpreted with caution.

### Steroid and bisphosphonate exposure

No significant differences in pulmonary function change were observed between patients with and without documented corticosteroid exposure during any study interval. However, only 2 patients had not received corticosteroids, resulting in markedly imbalanced groups and limited power to identify clinically meaningful differences. Therefore, no definitive conclusions can be drawn regarding the effects of corticosteroid exposure on postoperative pulmonary function in patients with DMD.

The comparison by bisphosphonate exposure is best understood as a difference in the timing of pulmonary decline rather than its extent. Patients with documented bisphosphonate exposure had a greater decrease in FVC and FVC percent predicted over the early postoperative interval, but no difference was present over either interval ending at final follow-up, and the two groups reached similar values at 2 years. None of these comparisons survived adjustment for multiplicity. Several sources of confounding are also present: bisphosphonate-exposed patients were operated on later in the series, were all corticosteroid-treated, and are by indication patients with established bone fragility, which may mark greater cumulative corticosteroid exposure or more advanced disease [4, 27]. No mechanism links bisphosphonate therapy to an acute postoperative fall in vital capacity, and we regard a period effect, confounding by indication, or chance as the more likely explanations. No prior studies have specifically examined bisphosphonate use and perioperative pulmonary outcomes in DMD.

### Limitations

This study has several limitations. The small sample size limited statistical power and increased the risk of both type I and type II error; the null correlations with FVC percent predicted in particular should be regarded as inconclusive rather than negative. The retrospective design introduced the potential for selection bias, missing data, and variation in the timing and technique of pulmonary testing. The absence of a nonsurgical control group and of serial preoperative pulmonary measurements prevented determination of whether surgery altered the natural rate of respiratory decline. FVC, MIP, and MEP are effort-dependent and may be influenced by cooperation, fatigue, pain, positioning, and testing technique. Height was measured at each spirometry visit, but in nonambulatory patients with contractures and spinal deformity any height measurement is an approximation of true length, and visit-to-visit variation in that measurement propagates directly into percent-predicted values; this is a plausible contributor to the greater variance of percent-predicted than of measured FVC in this cohort. Medication data were limited, particularly regarding the indication, timing, duration, and cumulative exposure to corticosteroids and bisphosphonates. Finally, the approximately 2-year pulmonary follow-up may not capture the longer-term respiratory trajectory after PSF.

## Conclusions

In this cohort of nonambulatory males with DMD, PSF for scoliosis was followed by early stability of FVC percent predicted and a significant decline by 2-year follow-up, consistent with the expected natural history of respiratory decline in DMD. Greater curve correction was not associated with better pulmonary outcomes: it bore no significant relationship to change in FVC percent predicted, and the association with measured FVC was a fixed perioperative difference that did not widen over follow-up and most likely reflects the severity of the preoperative deformity. Respiratory muscle pressures were stable over the study period, and the mechanism of the observed decline cannot be determined from these data. These findings do not support pulmonary preservation as an expected benefit of deformity correction, and they support framing the goals of PSF in DMD in terms of functional, postural, and quality-of-life benefits rather than respiratory preservation.

## Data Availability

No datasets were generated or analysed during the current study.
